# deepDriver: Predicting Cancer Driver Genes Based on Somatic Mutations Using Deep Convolutional Neural Networks

**DOI:** 10.3389/fgene.2019.00013

**Published:** 2019-01-29

**Authors:** Ping Luo, Yulian Ding, Xiujuan Lei, Fang-Xiang Wu

**Affiliations:** ^1^Division of Biomedical Engineering, University of Saskatchewan, Saskatoon, SK, Canada; ^2^School of Computer Science, Shaanxi Normal University, Xian, China; ^3^School of Mathematics and Statistics, Hainan Normal University, Haikou, China; ^4^Department of Mechanical Engineering, University of Saskatchewan, Saskatoon, SK, Canada; ^5^Department of Computer Science, University of Saskatchewan, Saskatoon, SK, Canada

**Keywords:** deep learning, convolutional neural networks, driver gene prediction, cancer mutations, gene similarity network

## Abstract

With the advances in high-throughput technologies, millions of somatic mutations have been reported in the past decade. Identifying driver genes with oncogenic mutations from these data is a critical and challenging problem. Many computational methods have been proposed to predict driver genes. Among them, machine learning-based methods usually train a classifier with representations that concatenate various types of features extracted from different kinds of data. Although successful, simply concatenating different types of features may not be the best way to fuse these data. We notice that a few types of data characterize the similarities of genes, to better integrate them with other data and improve the accuracy of driver gene prediction, in this study, a deep learning-based method (deepDriver) is proposed by performing convolution on mutation-based features of genes and their neighbors in the similarity networks. The method allows the convolutional neural network to learn information within mutation data and similarity networks simultaneously, which enhances the prediction of driver genes. deepDriver achieves AUC scores of 0.984 and 0.976 on breast cancer and colorectal cancer, which are superior to the competing algorithms. Further evaluations of the top 10 predictions also demonstrate that deepDriver is valuable for predicting new driver genes.

## 1. Introduction

Cancer is driven by various types of mutations, such as single nucleotide variants (SNVs), insertions or deletions (Indels) and structural variants. Identifying driver genes whose mutations cause cancer could help us decipher the mechanism of cancer, which is beneficial to the development of novel drugs and therapies.

With the advances in next-generation sequencing technologies, massive amounts of cancer genomic data have been published, which elevate the identification of driver genes. Currently, many computational methods have been proposed. Based on their rationale, existing methods can be divided into several types. A typical kind of methods is those based on the mutation frequency. These methods find “significantly mutated genes” (SMG) whose mutation rates are significantly higher than the background mutation rate and judge them as driver genes. For instance, OncodriveCLUST finds positions with mutation rates higher than the background mutation rate and predicts driver genes from clusters generated based on these seed positions (Tamborero et al., 2013). MutsigCV identifies SMGs by building a patient-specific background mutation model with gene expression data and DNA replication time data (Lawrence et al., 2014). However, due to the heterogeneity of tumors, constructing a reliable background mutation model is difficult (Cheng et al., 2015), which limits the performance of frequency-based methods. Another type of methods predicts driver genes by network analysis. For example, DawnRank predicts driver genes by ranking the genes in a gene interaction network (GIN) with PageRank algorithm (Hou and Ma, 2014). SCS uses network control strategy to find driver mutations that can drive the regulation network from the normal state to disease states (Guo et al., 2018). Considering that GINs are downloaded from online databases, such as BioGrid (Chatr-Aryamontri et al., 2017) and HPRD (Keshava Prasad et al., 2008), which contain many false positives, network-based methods need more accurate GIN to improve their prediction accuracy.

As the increasing number of experimentally validated driver genes, researchers start to use machine learning algorithms to predict new driver genes. These methods usually train a classifier with features characterizing the functional impact of mutations. For instance, CHASM trains a random forest classifier with 86 predictive features (Wong et al., 2011). CanDrA trains an SVM with 95 features obtained from 10 functional impact-based algorithms, such as SIFT (Kumar et al., 2009) and CHASM. Since the number of driver genes is much smaller than that of passenger genes, selecting gold-standard driver genes (positive data) and a set of high-quality nonfunctional passenger genes (negative data) is difficult for machine learning-based methods. However, with reasonable downsampling, these methods can also achieve better performance than other types of algorithms. Tokheim et al. propose a random forest algorithm (known as 20/20+) and compare it with seven classical driver gene prediction algorithms [ActiveDriver (Reimand and Bader, 2013), MuSiC (Dees et al., 2012), MutsigCV (Lawrence et al., 2014), OncodriveCLUST (Tamborero et al., 2013), OncodriveFM (Gonzalez-Perez and Lopez-Bigas, 2012), OncodriveFML (Mularoni et al., 2016) and TUSON (Davoli et al., 2013)] in Tokheim et al. (2016). Results show that 20/20+ performs best among the eight algorithms, which demonstrate that machine learning models are able to predict driver genes given the limited known driver-disease associations.

At present, most machine learning-based methods use random forest and SVM as the classifier. To improve the prediction accuracy, various kinds of features extracted from different types of data are used to train the classifier. Despite the increase of the dimensionality, simply concatenating all these features may not be the best approach to integrate different types of data. Considering that several types of data can be used to characterize the similarities of genes, if we construct similarity networks with these data and combine them with other predictive features, the prediction accuracy of the algorithms should be improved compared to that obtained from a simple feature concatenation. Thus, in this study, a deep learning-based method is proposed to predict driver genes by combining similarity networks with features that characterize the functional impact of mutations (deepDriver). Specifically, candidate driver genes are predicted by a convolutional neural network (CNN) trained with mutation-based feature matrix constructed based on the topological structure of a similarity network. The algorithm leverages the similarity of gene expression patterns and the functional impact of mutations simultaneously, which can better fuse these two types of data and improve the prediction accuracy. To our knowledge, this is the first time that CNN is combined with similarity network to predict driver genes.

In the rest of the paper, section 2 describes the materials and methods used in the study. Section 3 analyzes the results of the evaluation. Section 4 draws some conclusions.

## 2. Materials and Methods

### 2.1. General Model

CNN is successful in many areas, such as image classification and speech recognition. The key component of a CNN is the convolutional (CONV) layer, which helps the model to learn local and global structures from the input data. In an image classification problem, these structures include edges, curves, corners, etc. While in a driver gene prediction problem, traditional input data contain distinct features that characterize different properties of genes, which cannot be directly applied to CNN.

We notice that pixels in a small region share the same filters because they have similar grayscale. In a gene similarity network (GSN), genes and their neighbors also have similar properties. If we reconstruct the traditional input data with GSN so that features of similar genes are close to each other, CNN can then be applied to these reconstructed data. Instead of edges and curves learned from the images, topological structures of the similarity networks are learned by CNN with this strategy. In addition, the strategy allows CNN to learn the similarities of genes and the properties of the original input data simultaneously, which can improve the accuracy of driver gene prediction.

**Figure 1:**
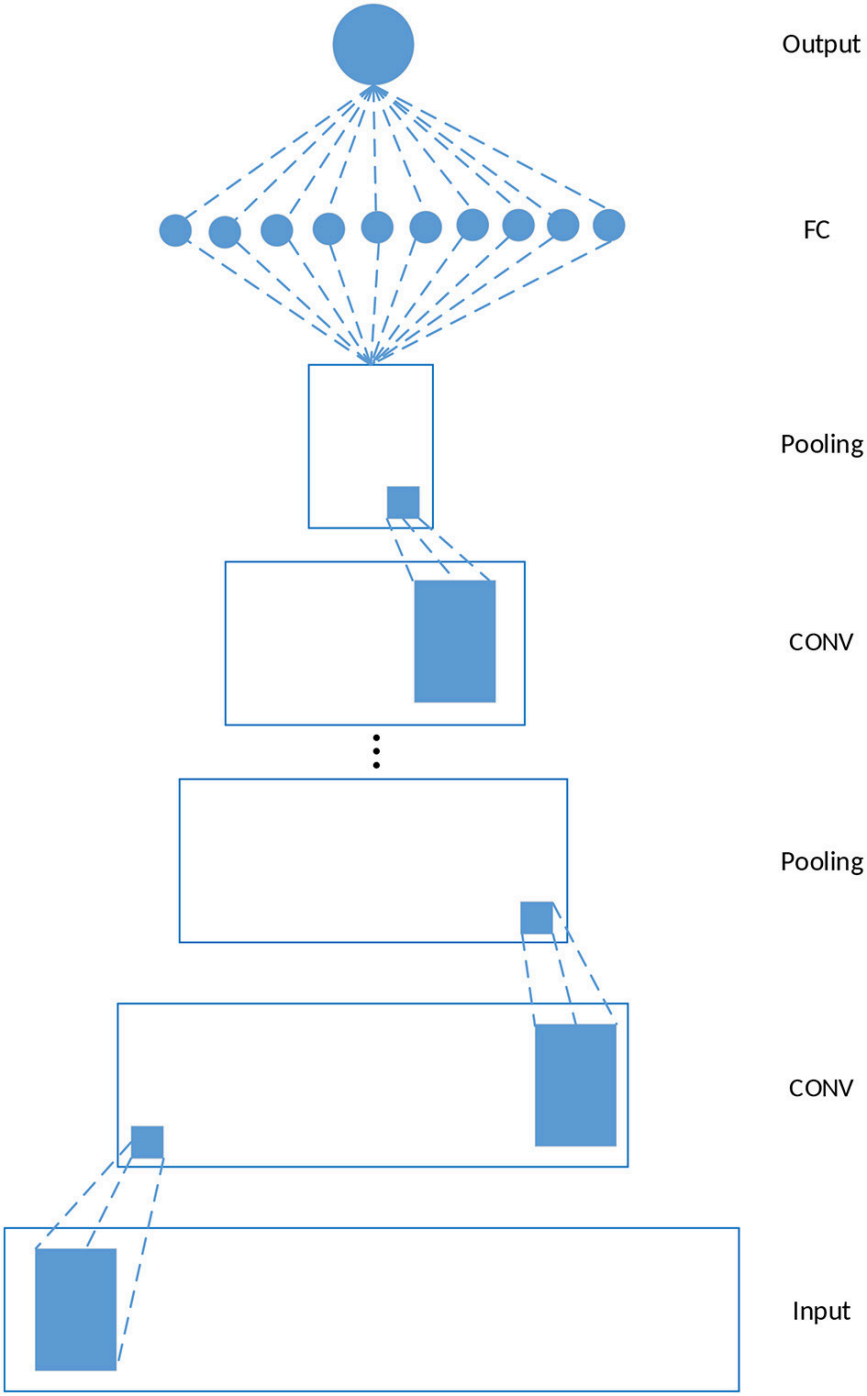
Schematic 1-D CNN. In this study, each CONV layer is followed by a pooling layer and the CONV-POOL pattern is repeated for several times. The final structure of the model is determined by grid search.

Figure 1 depicts a schematic example of a 1-dimensional CNN, which is used in our study. The model consists of five kinds of layers: Input layer, CONV layers, pooling layers, Fully-Connected (FC) layers, and Output layer. Given a feature matrix $\phi_{i}\in R^{2k\times n_{f}}$ constructed by the feature vectors of *g*_*i*_ and its *k* neighbors where *n*_*f*_ is the dimension of the feature vectors of *g*_*i*_, the output of a CONV layer corresponds to the input ϕ_*i*_ and the filter *w*_*j*_ is calculated as follows

(1) $$\begin{array}{r} A\left(i,j\right)=f\left(w_{j}\phi_{i}+b_{j}\right) \end{array}$$

where *b*_*j*_ denotes the bias corresponding to *w*_*j*_, *f* is an activation function which is ReLU in this study. *w*_*j*_ϕ_*i*_ is still the dot product of *w*_*j*_ and ϕ_*i*_ except that the calculation is restricted to be local spatially. Each CONV layer is followed by a pooling layer, and the CONV-POOL pattern is repeated for several times. The final structure of the model used for driver gene prediction is determined by grid search, and the results are discussed in section 3.2. The construction of ϕ_*i*_ is discussed in the next section.

### 2.2. Network-Based Convolution

The convolution is performed by combining mutation-based features with gene similarity networks. Many approaches can be used to calculate the similarities of genes. In this study, to characterize the relationships between genes in the disease states, Pearson correlation coefficient (PCC) defined by the following equation is used to calculate the similarities.

(2) $$\begin{array}{r} r\left(g_{i},g_{j}\right)=\frac{\sum_{q=1}^{v}\left(e_{iq}-{ē}_{i}\right)\left(e_{jq}-{ē}_{j}\right)}{\sqrt{\sum_{q=1}^{v}{\left(e_{iq}-{ē}_{i}\right)}^{2}}\sqrt{\sum_{q=1}^{v}{\left(e_{jq}-{ē}_{j}\right)}^{2}}} \end{array}$$

where **e**_*i*_ = (*e*_*i*1_, *e*_*i*2_, …, *e*_*iv*_) denotes the expression values of *g*_*i*_ in *v* tumor samples, and ${\bar{\text{e}}}_{i}$ is the mean of **e**_*i*_. An undirected network *N* is constructed by *k*-nearest neighbors (kNN) algorithm (Cover and Hart, 1967) in which every gene is connected to genes that have the *k* largest PCC scores with itself.

**Figure 2:**
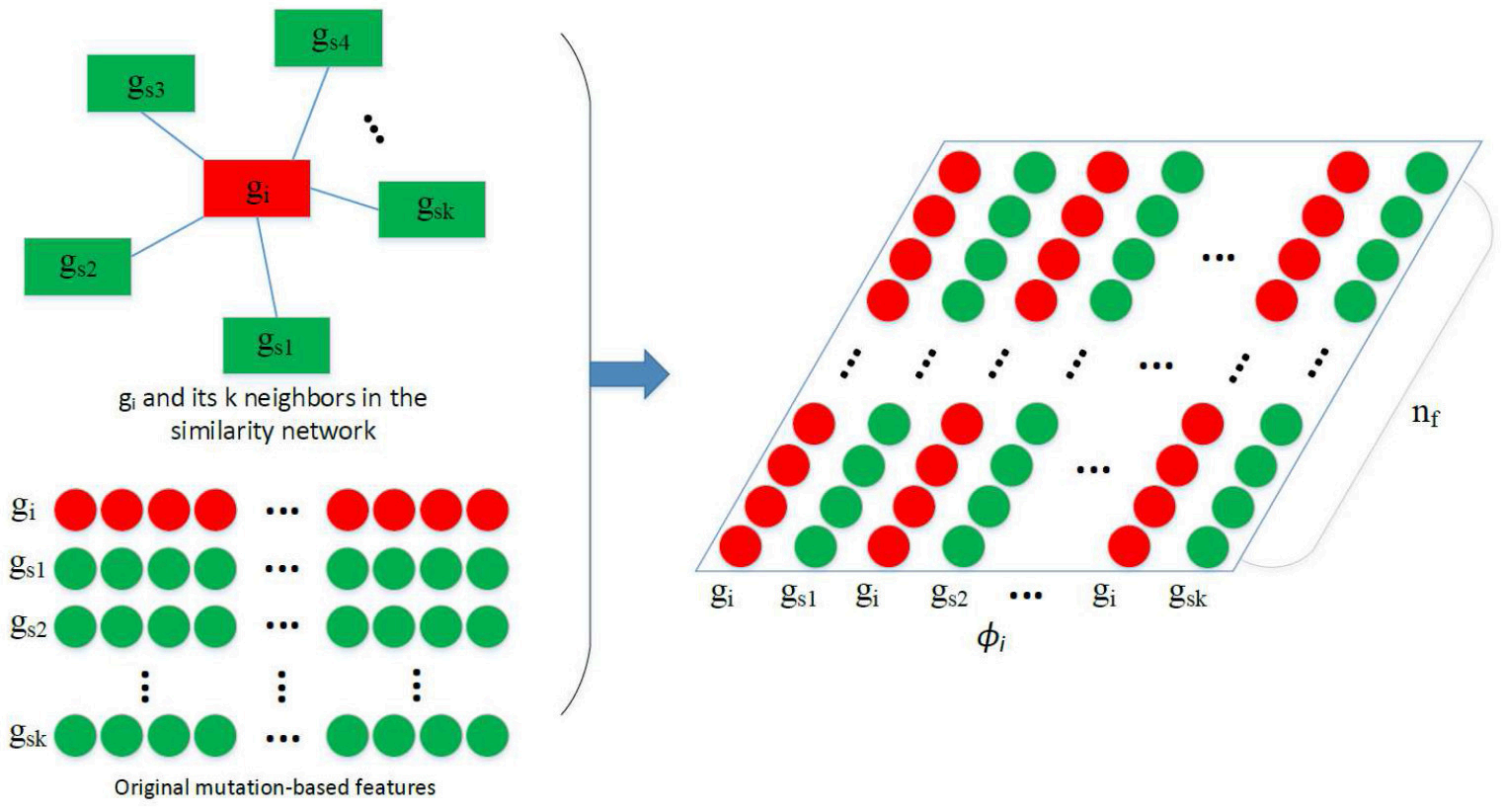
Construction of ϕ_*i*_. Given the feature vectors of *g*_*i*_ and its *k* nearest neighbors *g*_*s*1_, *g*_*s*2_, …, *g*_*sk*_, a feature matrix ϕ_*i*_ is constructed by arranging the 2*k* vectors into a 2*k* × *n*_*f*_ matrix, which is then used in the convolution.

After obtaining *N*, the construction of ϕ_*i*_ used in the convolution is depicted by Figure 2. Assuming we have obtained a feature vector *x*_*i*_ for each gene *g*_*i*_, and *g*_*s*1_, *g*_*s*2_, …, *g*_*sk*_ are the *k* nearest neighbors of *g*_*i*_ in *N*, where *pcc*(*g*_*i*_, *g*_*s*1_) > *pcc*(*g*_*i*_, *g*_*s*2_) > … > *pcc*(*g*_*i*_, *g*_*sk*_). Feature matrix $\phi_{i}\in R^{2k\times n_{f}}$ is built as depicted by the figure. In ϕ_*i*_, features of similar genes are close to each other so that they can share the same filters in the CONV layer.

### 2.3. Mutation-Based Features

For each gene of a specific disease, 12 features are extracted from the mutation datasets. Table 1 lists the names and descriptions of these features. Among them, the first eight ones measure the fraction of a specific type of mutation among all the mutations. The tenth and eleventh feature measure the rate of missense mutations and non-silent mutations to silent mutations, respectively. The last two features measure the positional clustering of different types of mutations and are calculated as follows

(3) $$\begin{array}{r} E_{i}=\frac{-\underset{i}{\sum }p_{j}\log_{2}p_{j}}{\log_{2}m} \end{array}$$

For the normalized missense entropy, *m* is the total number of missense mutations of *g*_*i*_, and *p*_*j*_ = κ_*j*_/*m* where κ_*j*_ is the number of missense mutations in the *j*-th codon. For the normalized mutation entropy, *m* is the total number of all types of mutations of *g*_*i*_. Different mutations are binned together based on their types, except for that missense mutations are binned based on their codon positions, different silent mutations are divided into their own bins. Inactivating mutations (nonsense, translation start site, nonstop, splice site) are grouped into a single bin.

**Table 1:** Twelve features extracted from mutation data.

**No**.	**Name**	**Description**
1	Silent fraction	Fraction of silent mutations
2	Nonsense fraction	Fraction of nonsense mutations
3	Splice site fraction	Fraction of splice site mutations
4	Missense fraction	Fraction of missense mutations
5	Recurrent missense fraction	Fraction of recurrent missense mutations
6	Frameshift indel fraction	Fraction of frameshift indel mutations
7	Inframe indel fraction	Fraction of inframe indel mutations
8	Lost start and stop fraction	Fraction of lost start and stop mutations
9	Missense to silent	Ratio of missense to silent mutations
10	Non-silent to silent	Ratio of non-silent to silent mutations
11	Normalized missense position entropy	See section 2.3
12	Normalized mutation entropy	See section 2.3

These 12 features have been used in many machining learning-based methods (Vogelstein et al., 2013; Tokheim et al., 2016). To demonstrate the superiority of our model, we did not use any other features proposed by specific methods. In addition, during the implementation of the competing methods (SVM, 20/20+), only these 12 features are used to train their models.

### 2.4. Data Sources

In this study, deepDriver was evaluated on three types of cancer: breast invasive carcinoma (BRCA), colon adenocarcinoma (COAD) and lung adenocarcinoma (LUAD). The mutation data and gene expression data of these three diseases were downloaded from the NCI Genomic Data Commons (GDC) (Grossman et al., 2016). For the mutation data, quality control was applied by filtering out hypermutated samples (> 1, 000 intragenic somatic variants) (Vogelstein et al., 2013). In total, 228,046, 168,746, and 287,667 somatic variants were obtained for BRCA, COAD, and LUAD, respectively. For gene expression, datasets of 1,102 BRCA, 478 COAD and 551 LUAD primary tumor samples measured by RNA-Seq were downloaded. We chose the data normalized by FPKM and converted the values to TPM by the method proposed in Pachter (2011). Three steps were then performed to remove the genes that are barely expressed in tumor samples. First, TPM values <1 were considered unreliable and replaced by 0. Second, log_2_(TPM+1) was applied to all TPM values. Third, genes expressed in < 10% of all tumor samples were removed.

Gene ids were standardized to the gene names provided by HUGO Gene Nomenclature Committee (downloaded Aug 1, 2018) (Yates et al., 2016). Only genes that have both mutation and expression data are kept. Finally, 13,777 genes for BRCA, 11,282 genes for COAD, and 13,731 genes for LUAD passed the quality control.

The driver genes were collected from two sources—the Cancer Gene Census category (CGC) (Forbes et al., 2016) and the genes published in Bailey et al. (2018). Genes in CGC were divided into two tiers, and we used genes in Tier 1 as driver genes because strong evidence has proved their oncogenic role in cancer genesis. It is of note that both oncogene and tumor suppressor gene (TSG) are regarded as driver gene in this study. In total, 37 driver genes for BRCA, 42 driver genes for COAD and 12 driver genes for LUAD were collected from CGC. The Bailey et al.'s dataset (Bailey et al., 2018) contains 299 driver genes associated with 33 types of cancer. In total, 29 driver genes for BRCA, 20 driver genes for COAD and 20 driver genes for LUAD were collected.

To validate the performance of the algorithm, the structure of the model was first determined by the grid search using the driver genes of BRCA and COAD collected from CGC. Then, the optimal model was directly applied to LUAD without fine-tuning the hyperparameters. Similarly, when the model was trained with the driver genes published in Bailey et al. (2018), the optimal hyperparameters were used without fine-tuning.

### 2.5. Evaluation Metrics

The algorithm was evaluated in two steps. In the first step, deepDriver was compared with 20/20+ and SVM in terms of the AUC (area under the receiver operating characteristic (ROC) curve) scores obtained from 10-fold cross-validation. ROC curve plots the false positive rate (FPR) against the true positive rate (TPR) at different thresholds. FPR and TPR are defined as follows

(4) $$\begin{array}{l} FPR=\frac{FP}{FP+TN}\\ TPR=\frac{TP}{TP+FN} \end{array}$$

where *TP*, *FP*, *TN*, and *FN* are the numbers of true positives, false positives, true negatives, and false negatives, respectively. In this study, a true positive is a driver gene predicted as a driver gene, a false positive is a passenger gene predicted as driver gene, a true negative is a passenger gene predicted as a passenger gene, and a false negative is a driver gene predicted as a passenger gene. Algorithm with the highest AUC score performs the best.

Since the number of passenger genes is much larger than that of the driver genes, a method is needed to solve the imbalanced issue. Currently, two types of methods can be used to solve the imbalanced problem: data level methods and classifier level methods (Buda et al., 2018). In this study, a data level method, downsampling, was used to reduce the size of the passenger genes. Specifically, a subset of passenger genes was randomly selected from all the passengers so that the numbers of positive samples (driver genes) and negative samples (passenger genes) are equal. This approach was run for five times which generated five sets of data. During the cross-validation, for each set of data, all the positive and negative samples were randomly split into ten groups, and the CNN model was validated for ten rounds. In each round, one group of samples were used as the testing data while the rest nine groups of samples were used as the training data.

Additionally, since passenger genes are barely reported in existing literature, in this study, genes that have not been reported as cancer driver genes (unknown genes) were regarded as passenger genes. This strategy was used because of the following two reasons. First, the numbers of the selected passenger genes and the undiscovered driver genes are both much less than that of the unknown genes. Potential driver genes only have a small change to be selected as passenger genes (Davoli et al., 2013). Second, the final results were obtained by taking the average predictions of the five sets of data. This bagging strategy would improve the stability and accuracy of the results and reduce the impact of a potential driver gene selected as a passenger gene. Finally, the 10-fold cross-validation was run for five times for each dataset to reduce the influence of random shuffling, and the average AUC score was used to evaluate the performance of the algorithms.

In the second step, all the unknown genes were ranked by their probabilities of being driver genes, and the top 10 predictions were searched from the existing literature to check whether our predictions are in concert with existing studies. We also ranked the unknown genes by SVM, 20/20+ and OncodriveCLUST and compared their results with those of deepDriver in terms of the number of genes having been analyzed in existing literature.

### 2.6. Implementation

The algorithm was implemented using Keras (Chollet, 2015) with TensorFlow (Abadi et al., 2015) as the backend engine. We have tested the program on both CPU and GPU versions of TensorFlow and the model can be efficiently trained with or without the help of GPU. A reference implementation is available at GitHub.

## 3. Results

### 3.1. Hyperparameters

In this study, the architecture of CNN is determined by the following hyperparameters.

1. The number of the CONV layers (*ncl*)
2. The number of the FC layers (*nfl*)
3. The number of the nodes in the CONV layers (*ncn*)
4. The number of the nodes in the FC layers (*nfn*)

These hyperparameters were determined by grid search, with *ncl* searched from {1, 2, 3, 4}, *nfl* searched from {1, 2, 3}, *ncn* searched from {12, 24, 48} and *nfn* searched from {24, 48, 96}. The optimal values of *ncl, nfl, ncn*, and *nfn* are 2, 1, 24, and 48, respectively. In addition, zero padding was used in the CONV layers except the first one. The size of the filters, the window size of the pooling layers and the stride sizes used in the CONV layers and the pooling layers were all empirically set to 2.

The number of neighbors used by kNN algorithms was also determined by grid search. We searched *k* from {3, 5, 7, 9, 11, 13, 15}, and finally, *k* = 9 and *k* = 7 were chosen for BRCA and COAD, respectively. In fact, the AUC scores were all above 0.950 when 7 ≤ *k* ≤ 15. Based on our previous study, *k* = 7 is enough to generate high-quality similarity networks (Luo et al., 2017). Thus, *k* = 7 was used when the dataset of LUAD was analyzed by our deepDriver. Meanwhile, for other types of cancer not discussed in this study, *k* = 7 is also recommended when the similarity network is constructed.

For 20/20+, a random forest of 200 trees was used based on the suggestions of Tokheim et al. (2016). For SVM, the model was implemented with a linear kernel and RBF kernel. The penalty parameter *C* was searched from {0.1, 0.01, 0.001, 1, 10, 100, 1,000}, and γ was searched from {1/12, 0.001, 0.0001, 0.00001}. Finally, for BRCA and COAD, SVM performed the best with an RBF kernel, when *C* = 1, γ = 0.0001; for LUAD, SVM performed the best with an RBF kernel, when *C* = 1, 000, γ = 0.00001.

### 3.2. Cross-Validation

**Figure 3:**
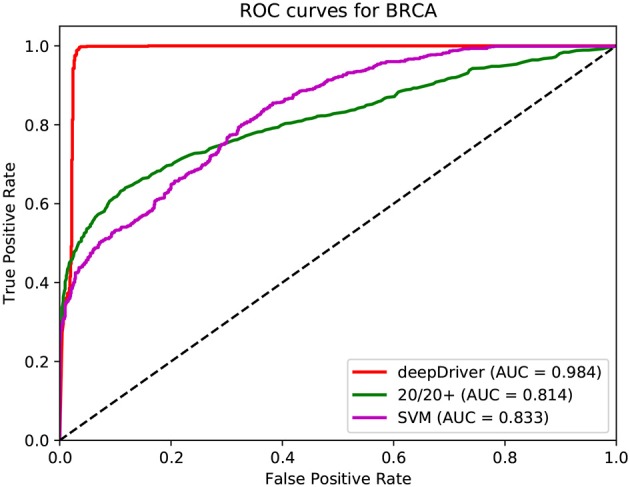
ROC curves of the three algorithms obtained on the dataset of BRCA. The red, green, and magenta lines depict the ROC curves of deepDriver, 20/20+ and SVM, respectively. The AUC value of deepDriver is 0.984, which is at least 15.1% higher than that of the other two algorithms.

**Figure 4:**
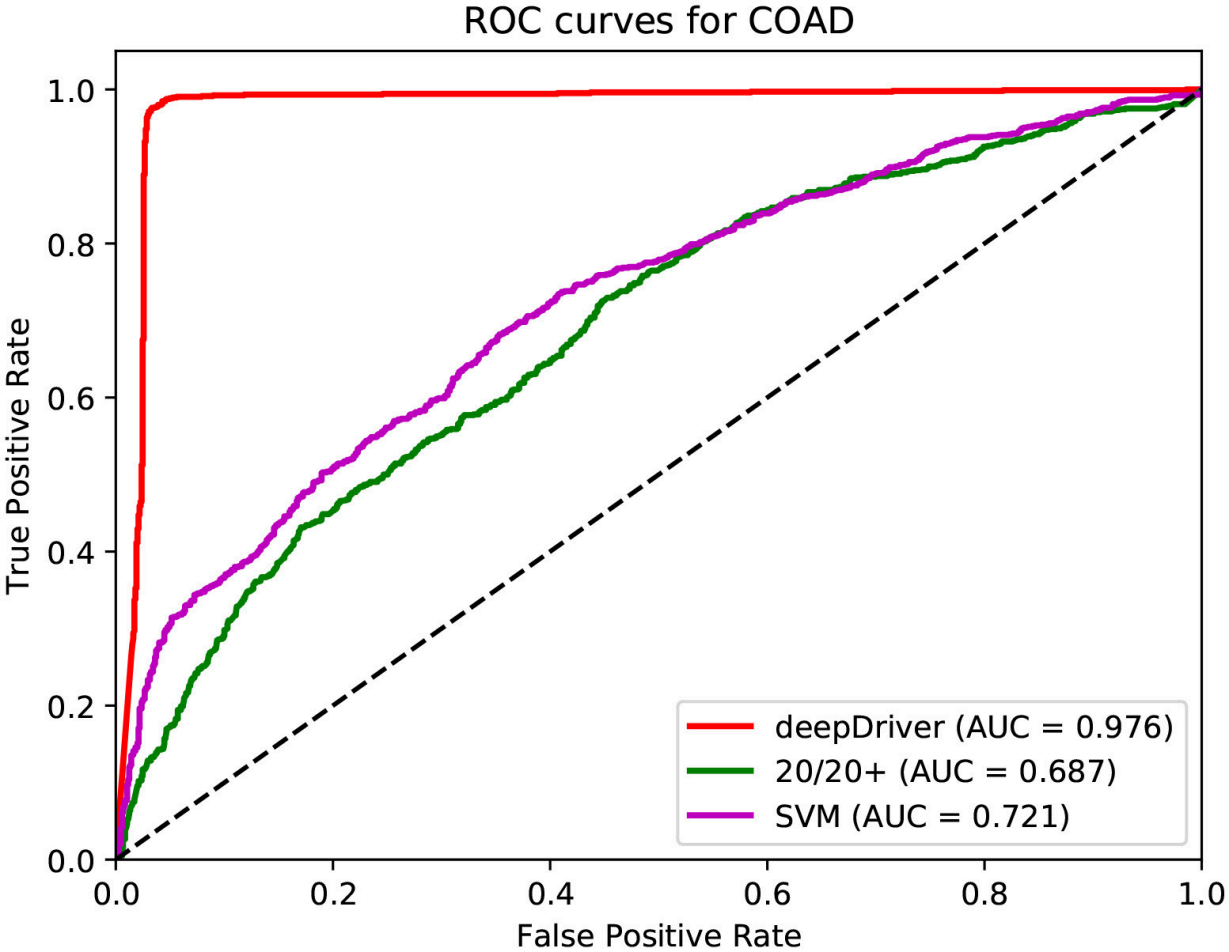
ROC curves of the three algorithms obtained on the dataset of COAD. The red, green, and magenta lines depict the ROC curves of deepDriver, 20/20+ and SVM, respectively. The AUC value of deepDriver is 0.976, which is at least 25.5% higher than that of the other two algorithms.

**Figure 5:**
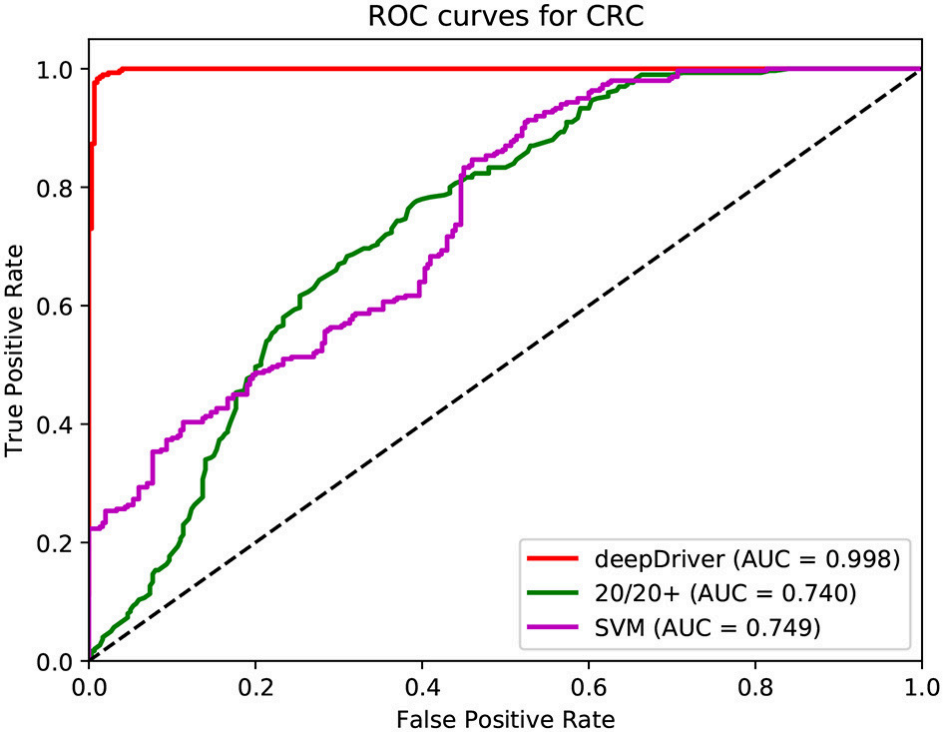
ROC curves of the three algorithms obtained on the dataset of LUAD. The red, green, and magenta lines depict the ROC curves of deepDriver, 20/20+ and SVM, respectively. The AUC value of deepDriver is 0.998, which is at least 24.9% higher than that of the other two algorithms.

Figures 3–5 show the results of the ROC curves and the corresponding AUC scores of deepDriver, 20/20+ and SVM on BRCA, COAD and LUAD, respectively. According to the figures, deepDriver achieved AUC scores of 0.984, 0.976, and 0.998 on BRCA, COAD, and LUAD, respectively, which were at least 15.1% higher than those of the two competing algorithms, especially for COAD and LUAD where the AUC scores of the competing algorithms were <0.750.

**Figure 6:**
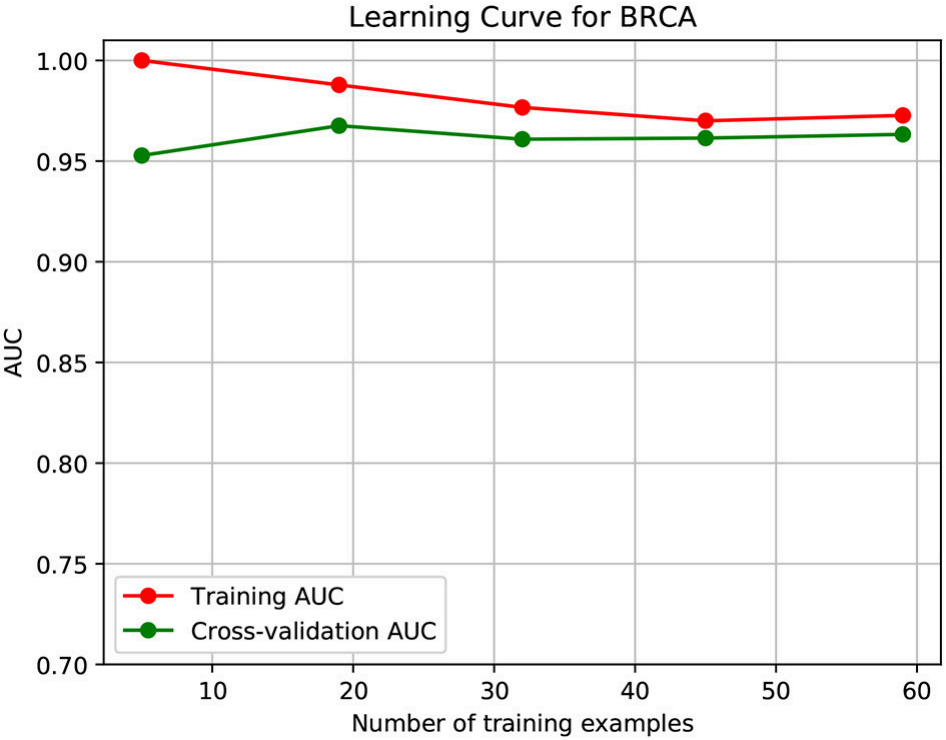
Learning curve for BRCA.

**Figure 7:**
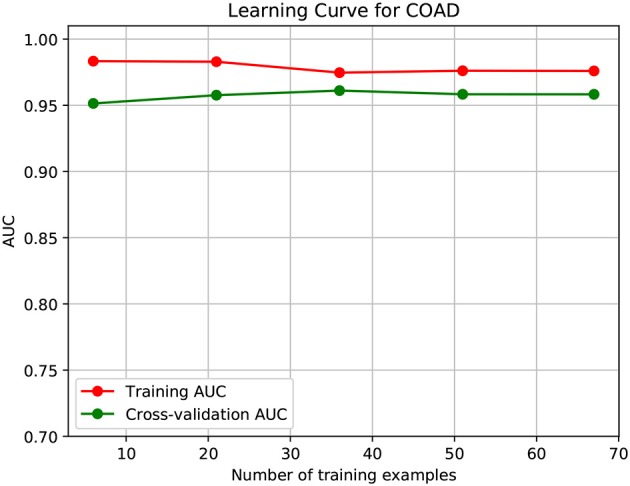
Learning curve for COAD.

**Figure 8:**
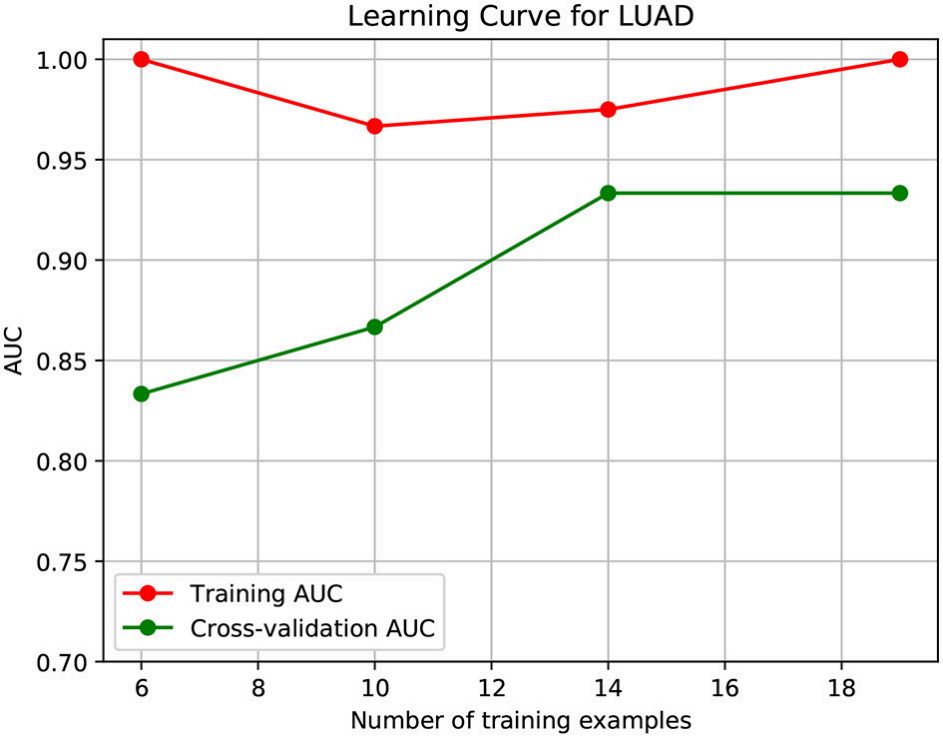
Learning curve for LUAD.

To further demonstrate that the model was not overfitted, the learning curves were plotted using the datasets of the three types of cancer. For each type of cancer, 80% of the total samples were used as training data while the rest 20% samples were left to test the performance of the model. Figures 6–8 show the results of the learning curves. The AUC scores obtained from the testing set improved with the increase of the number of the training samples, which demonstrates that the model is not overfitted. In the meantime, the AUC scores obtained with a small amount of samples also demonstrate that the model is able to produce meaningful results even if the number of the known driver genes is <10.

**Figure 9:**
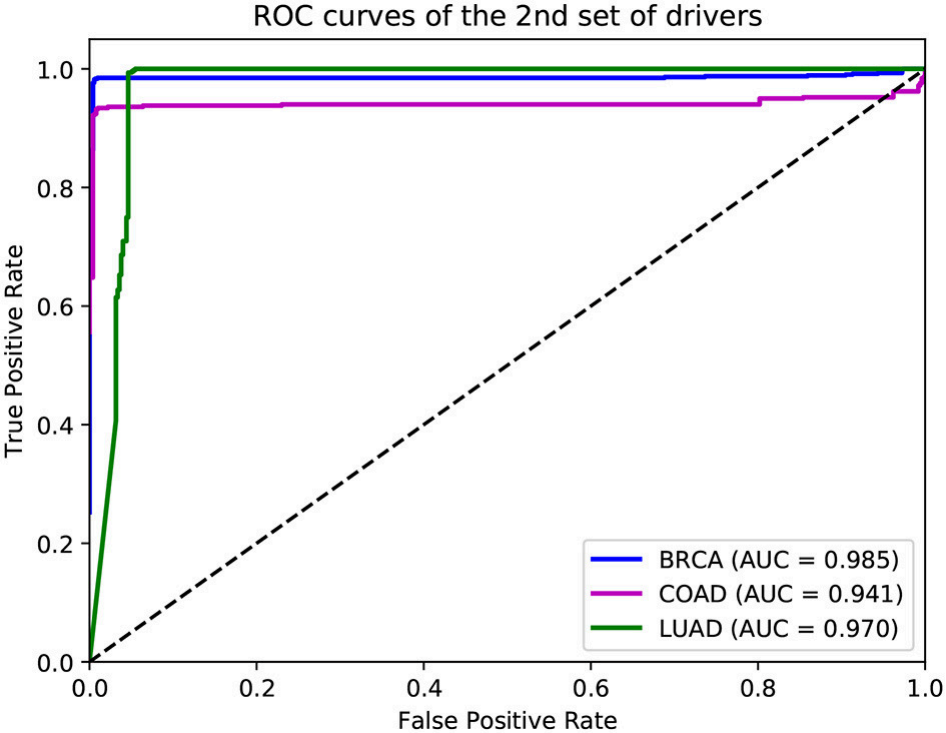
ROC curves of deepDriver obtained from the second sets of driver genes.

In addition to the driver genes collected from CGC, our deepDriver was also validated using the driver genes published in Bailey et al. (2018). As discussed in section 2.4, the optimal hyperparameters obtained from the first set of drivers were directly used to evaluate the model. Figure 9 depicts the resulted ROC curves. Our deepDriver obtained AUC scores of 0.985, 0.941, and 0.970 on BRCA, COAD, and LUAD, respectively.

### 3.3. *De novo* Study

To further evaluate the performance of deepDriver, the unknown genes were ranked by their probabilities of being driver genes predicted by the model. Similar to the cross-validation, 5 sets of data were used to train the model and the unknown genes were ranked by the average probabilities. Meanwhile, we also ranked the unknown genes using the three competing algorithms and compared their results with those of deepDriver in terms of the number of genes that have been studied as drivers in existing literature.

Table 2 shows the top 10 predicted driver genes of deepDriver. Six out of the 10 genes have been studied in existing literature or databases as potential driver genes of BRCA. The ninth gene “DST” was found to have the potential to drive ductal carcinoma *in situ* to breast cancer (Lee et al., 2012). Five out of the 10 genes have been studied as driver genes of COAD in the existing literature. Meanwhile, among the rest 5 genes, “AMER1” and “ADAMTSL3” were found to be frequently mutated in COAD (Koo et al., 2007; Sanz-Pamplona et al., 2015). “LAMA3” were predicted as biomarkers which could be used to diagnose COAD in the early stage (Choi et al., 2015). “KMT2A” belongs to the KMT2 family which is related to COAD (Rao and Dou, 2015). Four out of 10 genes have been studied as driver genes of LUAD. The tenth gene “HERC2P3” contains a microsatellite locus that can precisely discriminate LUAD samples and non-tumor samples (Velmurugan et al., 2017). As for three competing algorithms, Tables 3–5 show their prediction results. In summary, deepDriver performed better than the three competing algorithms in predicting new cancer drivers. Its prediction results were in concert with existing studies which further reveal the value of deepDriver in predicting cancer driver genes.

**Table 2:** Top 10 predictions of deepDriver.

**Gene names**	**References**
**BRCA**	
PTEN	Kechagioglou et al., 2014
HCFC1	Gonzalez-Perez et al., 2013; Rubio-Perez et al., 2015
UTRN	Cornen et al., 2014
ZNF517	
STAG2	Gonzalez-Perez et al., 2013; Rubio-Perez et al., 2015
ZFP36L1	Loh et al., 2017
ZNF91	
VPS13C	
DST	
FBXW7	Cao et al., 2016
**COAD**	
AMER1	
SOX9	Prévostel and Blache, 2017
NRAS	Meriggi et al., 2014
MTOR	Wang and Zhang, 2014
ATM	AlDubayan et al., 2018
ADAMTSL3	
ELMO1	Zheng et al., 2017
TG	
LAMA3	
KMT2A	
**LUAD**	
XIST	Wang et al., 2017
MALAT1	Li et al., 2018
STK11	Pécuchet et al., 2017
USH1C	
HSP90AB2P	
BNIP3P1	
EEF1A1P9	
UBE2MP1	
SMAD4	Haeger et al., 2016
HERC2P3	

**Table 3:** Top 10 predictions of 20/20+.

**Gene names**	**References**
**BRCA**	
KMT2C	Gala et al., 2018
PTEN	Kechagioglou et al., 2014
ANKRD12	
NF1	Uusitalo et al., 2017
ANKHD1-EIF4EBP3	
ARID4B	
MCM7	
MYO6	
MLLT4	Gonzalez-Perez et al., 2013
CEP128	
**COAD**	
ATM	AlDubayan et al., 2018
SOX9	Prévostel and Blache, 2017
LAMA3	
ADAMTSL3	
ELMO1	Zheng et al., 2017
OLFM1	
BRINP1	
ACVR1B	
CNOT1	
PCDH7	
**LUAD**	
LRRIQ1	
HECTD4	
EPB41L3	Kikuchi et al., 2005
NF1	Redig et al., 2016
CEP350	
PRKDC	
APC	
MYH9	
POSTN	
FN1	

**Table 4:** Top 10 predictions of SVM.

**Gene names**	**References**
**BRCA**	
VPS13C	
UTRN	Cornen et al., 2014
HCFC1	Gonzalez-Perez et al., 2013; Rubio-Perez et al., 2015
MLLT4	Gonzalez-Perez et al., 2013
ZNF91	
STAG2	Gonzalez-Perez et al., 2013; Rubio-Perez et al., 2015
FBXW7	Cao et al., 2016
MALAT1	
NRK	
BAZ2B	
**COAD**	
ATM	AlDubayan et al., 2018
NRAS	Meriggi et al., 2014
MTOR	Wang and Zhang, 2014
SOX9	Prévostel and Blache, 2017
ADAMTSL3	
ELMO1	Zheng et al., 2017
AMER1	
KMT2B	
FBN2	
KMT2A	
**LUAD**	
XIST	Wang et al., 2017
MALAT1	Li et al., 2018
USH1C	
SNRPN	
STK11	Pécuchet et al., 2017
SMAD4	Haeger et al., 2016
POLA1	
MAGEE1	
BRAF	
CTNNB1	

**Table 5:** Top 10 predictions of OncodriveCLUST.

**Gene names**	**References**
**BRCA**	
ACTN4	Honda, 2015
AFF2	
ATP2B3	
AVPR1B	
CASR	
CMYA5	
DIS3L	
EPB41L2	
FBXW8	
KCND3	
**COAD**	
AKAP12	He et al., 2018
C3orf20	
COL1A2	Yu et al., 2018
DOK1	Friedrich et al., 2016
FNDC1	
MSRB3	
NCOA2	Yu et al., 2016
NPHS1	
NRAP	
PCDHB13	

## 4. Conclusion

In this study, we proposed an algorithm to predict cancer driver genes with CNN. The method combined CNN with similarity networks so that the functional impact of mutations and similarities of gene expression can be learned simultaneously, which improve the accuracy of driver gene prediction. Experiments performed on BRCA, COAD, and LUAD then showed that deepDriver was superior to the competing algorithms in terms of both cross-validation and *de novo* prediction.

In the future, similarity networks calculated by different strategies and predictive features extracted by other algorithms can both be used to improve the prediction accuracy. Meanwhile, the algorithm can be applied to the pancancer dataset to predict generic cancer driver genes. Since the total number of cancer driver genes is much higher than that of a specific type of cancer, candidate driver genes can also be further classified into TSG and oncogene on the pancancer dataset.

## Author Contributions

F-XW conceived this study. F-XW, PL, YD, and XL discussed about the methods. PL implemented the algorithm, designed and performed the experiments. PL and F-XW wrote the manuscript. All authors read and approved the final manuscript.

### Conflict of Interest Statement

The authors declare that the research was conducted in the absence of any commercial or financial relationships that could be construed as a potential conflict of interest.

## Nomenclature

### Resource Identification Initiative

Genomic Data Commons Data Portal (GDC Data Portal), RRID:SCR_014514

COSMIC-Catalog Of Somatic Mutations In Cancer, RRID:SCR_002260

HGNC, RRID:SCR_002827

tensorflow, RRID:SCR_016345

## References

[B1] Abadi M. Agarwal A. Barham P. Brevdo E. Chen Z. Citro C. (2015). TensorFlow: Large-Scale Machine Learning on Heterogeneous Systems. Available online at: https://www.tensorflow.org

[B2] AlDubayan S. H. Giannakis M. Moore N. D. Han G. C. Reardon B. Hamada T.. (2018). Inherited dna-repair defects in colorectal cancer. Am. J. Hum. Genet. 102, 401–414. 10.1016/j.ajhg.2018.01.018 29478780 PMC5985280

[B3] Bailey M. H. Tokheim C. Porta-Pardo E. Sengupta S. Bertrand D. Weerasinghe A.. (2018). Comprehensive characterization of cancer driver genes and mutations. Cell 173, 371–385. 10.1016/j.cell.2018.02.060 29625053 PMC6029450

[B4] Buda M. Maki A. Mazurowski M. A. (2018). A systematic study of the class imbalance problem in convolutional neural networks. Neural Netw. 106, 249–259. 10.1016/j.neunet.2018.07.011 30092410

[B5] Cao J. Ge M.-H. Ling Z.-Q. (2016). Fbxw7 tumor suppressor: a vital regulator contributes to human tumorigenesis. Medicine 95:e2496. 10.1097/MD.0000000000002496 26886596 PMC4998596

[B6] Chatr-Aryamontri A. Oughtred R. Boucher L. Rust J. Chang C. Kolas N. K.. (2017). The biogrid interaction database: 2017 update. Nucleic Acids Res. 45, D369–D379. 10.1093/nar/gkw1102 27980099 PMC5210573

[B7] Cheng F. Zhao J. Zhao Z. (2015). Advances in computational approaches for prioritizing driver mutations and significantly mutated genes in cancer genomes. Brief. Bioinformatics 17, 642–656. 10.1093/bib/bbv068 26307061 PMC4945827

[B8] Choi M. R. An C. H. Yoo N. J. Lee S. H. (2015). Laminin gene lamb 4 is somatically mutated and expressionally altered in gastric and colorectal cancers. Apmis 123, 65–71. 10.1111/apm.12309 25257191

[B9] Chollet F. (2015). Keras. Available online at: https://keras.io

[B10] Cornen S. Guille A. Adélaïde J. Addou-Klouche L. Finetti P. Saade M.-R.. (2014). Candidate luminal b breast cancer genes identified by genome, gene expression and dna methylation profiling. PLoS ONE 9:e81843. 10.1371/journal.pone.0081843 24416132 PMC3886975

[B11] Cover T. Hart P. (1967). Nearest neighbor pattern classification. IEEE Trans. Inf. Theory 13, 21–27. 10.1109/TIT.1967.1053964

[B12] Davoli T. Xu A. W. Mengwasser K. E. Sack L. M. Yoon J. C. Park P. J.. (2013). Cumulative haploinsufficiency and triplosensitivity drive aneuploidy patterns and shape the cancer genome. Cell 155, 948–962. 10.1016/j.cell.2013.10.011 24183448 PMC3891052

[B13] Dees N. D. Zhang Q. Kandoth C. Wendl M. C. Schierding W. Koboldt D. C.. (2012). Music: identifying mutational significance in cancer genomes. Genome Res. 22, 1589–1598. 10.1101/gr.134635.111 22759861 PMC3409272

[B14] Forbes S. A. Beare D. Boutselakis H. Bamford S. Bindal N. Tate J.. (2016). Cosmic: somatic cancer genetics at high-resolution. Nucleic Acids Res. 45, D777–D783. 10.1093/nar/gkw1121 27899578 PMC5210583

[B15] Friedrich T. Söhn M. Gutting T. Janssen K.-P. Behrens H.-M. Röcken C.. (2016). Subcellular compartmentalization of docking protein-1 contributes to progression in colorectal cancer. EBioMedicine 8, 159–172. 10.1016/j.ebiom.2016.05.003 27428427 PMC4919572

[B16] Gala K. Li Q. Sinha A. Razavi P. Dorso M. Sanchez-Vega F.. (2018). Kmt2c mediates the estrogen dependence of breast cancer through regulation of erα enhancer function. Oncogene 37, 4692–4710. 10.1038/s41388-018-0273-5 29755131 PMC6107480

[B17] Gonzalez-Perez A. Lopez-Bigas N. (2012). Functional impact bias reveals cancer drivers. Nucleic Acids Res. 40, e169–e169. 10.1093/nar/gks743 22904074 PMC3505979

[B18] Gonzalez-Perez A. Perez-Llamas C. Deu-Pons J. Tamborero D. Schroeder M. P. Jene-Sanz A.. (2013). Intogen-mutations identifies cancer drivers across tumor types. Nat. Methods 10, 1081. 10.1038/nmeth.2642 24037244 PMC5758042

[B19] Grossman R. L. Heath A. P. Ferretti V. Varmus H. E. Lowy D. R. Kibbe W. A.. (2016). Toward a shared vision for cancer genomic data. New Engl. J. Med. 375, 1109–1112. 10.1056/NEJMp1607591 27653561 PMC6309165

[B20] Guo W.-F. Zhang S.-W. Liu L.-L. Liu F. Shi Q.-Q. Zhang L.. (2018). Discovering personalized driver mutation profiles of single samples in cancer by network control strategy. Bioinformatics 34, 1893–1903. 10.1093/bioinformatics/bty006 29329368

[B21] Haeger S. M. Thompson J. J. Kalra S. Cleaver T. G. Merrick D. Wang X.-J.. (2016). Smad4 loss promotes lung cancer formation but increases sensitivity to dna topoisomerase inhibitors. Oncogene 35:577. 10.1038/onc.2015.112 25893305 PMC4615192

[B22] He P. Li K. Li S.-B. Hu T.-T. Guan M. Sun F.-Y.. (2018). Upregulation of akap12 with hdac3 depletion suppresses the progression and migration of colorectal cancer. Int. J. Oncol. 52, 1305–1316. 10.3892/ijo.2018.4284 29484387

[B23] Honda K. (2015). The biological role of actinin-4 (actn4) in malignant phenotypes of cancer. Cell Biosci. 5:41. 10.1186/s13578-015-0031-0 26288717 PMC4539665

[B24] Hou J. P. Ma J. (2014). Dawnrank: discovering personalized driver genes in cancer. Genome Med. 6:56. 10.1186/s13073-014-0056-8 25177370 PMC4148527

[B25] Kechagioglou P. Papi R. M. Provatopoulou X. Kalogera E. Papadimitriou E. Grigoropoulos P.. (2014). Tumor suppressor pten in breast cancer: heterozygosity, mutations and protein expression. Anticancer Res. 34, 1387–1400. 24596386

[B26] Keshava Prasad T. Goel R. Kandasamy K. Keerthikumar S. Kumar S. Mathivanan S.. (2008). Human protein reference database 2009 update. Nucleic Acids Res. 37(Suppl. 1):D767–D772. 10.1093/nar/gkn892 18988627 PMC2686490

[B27] Kikuchi S. Yamada D. Fukami T. Masuda M. Sakurai-Yageta M. Williams Y. N. (2005). Promoter methylation of dal-1/4.1 b predicts poor prognosis in non–small cell lung cancer. Clin. Cancer Res. 11, 2954–2961. 10.1158/1078-0432.CCR-04-2206 15837747

[B28] Koo B.-H. Hurskainen T. Mielke K. Aung P. P. Casey G. Autio-Harmainen H.. (2007). Adamtsl3/punctin-2, a gene frequently mutated in colorectal tumors, is widely expressed in normal and malignant epithelial cells, vascular endothelial cells and other cell types, and its mrna is reduced in colon cancer. Int. J. Cancer 121, 1710–1716. 10.1002/ijc.22882 17597111

[B29] Kumar P. Henikoff S. Ng P. C. (2009). Predicting the effects of coding non-synonymous variants on protein function using the sift algorithm. Nat. Protoc. 4:1073. 10.1038/nprot.2009.86 19561590

[B30] Lawrence M. S. Stojanov P. Mermel C. H. Robinson J. T. Garraway L. A. Golub T. R.. (2014). Discovery and saturation analysis of cancer genes across 21 tumour types. Nature 505:495. 10.1038/nature12912 24390350 PMC4048962

[B31] Lee S. Stewart S. Nagtegaal I. Luo J. Wu Y. Colditz G.. (2012). Differentially expressed genes regulating the progression of ductal carcinoma in situ to invasive breast cancer. Cancer Res. 72, 4574–4586. 10.1158/0008-5472.CAN-12-0636 22751464 PMC3899801

[B32] Li S. Mei Z. Hu H.-B. Zhang X. (2018). The lncrna malat1 contributes to non-small cell lung cancer development via modulating mir-124/stat3 axis. J. Cell. Physiol. 233, 6679–6688. 10.1002/jcp.26325 29215698 PMC13482422

[B33] Loh X. Y. Ding L. W. Koeffler H. P. (2017). Tumor suppressive role of ZFP36L1 by suppressing HIF1α and Cyclin D1 in bladder and breast cancer, in AACR Annual Meeting 2017 (Washington, DC: AACR). 10.1158/1538-7445.AM2017-4494

[B34] Luo P. Tian L.-P. Ruan J. Wu F.-X. (2017). Disease gene prediction by integrating ppi networks, clinical rna-seq data and omim data, in IEEE/ACM Transactions on Computational Biology and Bioinformatics (Shenzhen). 10.1109/TCBB.2017.2770120 29990218

[B35] Meriggi F. Vermi W. Bertocchi P. Zaniboni A. (2014). The emerging role of nras mutations in colorectal cancer patients selected for anti-egfr therapies. Rev. Recent Clin. Trials 9, 8–12. 10.2174/1568026614666140423121525 24758538

[B36] Mularoni L. Sabarinathan R. Deu-Pons J. Gonzalez-Perez A. López-Bigas N. (2016). Oncodrivefml: a general framework to identify coding and non-coding regions with cancer driver mutations. Genome Biol. 17:128. 10.1186/s13059-016-0994-0 27311963 PMC4910259

[B37] Pachter L. (2011). Models for transcript quantification from rna-seq. arXiv[Preprint].arXiv:1104.3889

[B38] Pécuchet N. Laurent-Puig P. Mansuet-Lupo A. Legras A. Alifano M. Pallier K.. (2017). Different prognostic impact of stk11 mutations in non-squamous non-small-cell lung cancer. Oncotarget 8:23831. 10.18632/oncotarget.6379 26625312 PMC5410347

[B39] Prévostel C. Blache P. (2017). The dose-dependent effect of sox9 and its incidence in colorectal cancer. Eur. J. Cancer 86, 150–157. 10.1016/j.ejca.2017.08.037 28988015

[B40] Rao R. C. Dou Y. (2015). Hijacked in cancer: the kmt2 (mll) family of methyltransferases. Nat. Rev. Cancer 15:334. 10.1038/nrc3929 25998713 PMC4493861

[B41] Redig A. J. Capelletti M. Dahlberg S. E. Sholl L. M. Mach S. L. Fontes C.. (2016). Clinical and molecular characteristics of nf1 mutant lung cancer. Clin. Cancer Res. 22, 3148–3156. 10.1158/1078-0432.CCR-15-2377 26861459 PMC5129179

[B42] Reimand J. Bader G. D. (2013). Systematic analysis of somatic mutations in phosphorylation signaling predicts novel cancer drivers. Mol. Syst. Biol. 9:637. 10.1038/msb.2012.68 23340843 PMC3564258

[B43] Rubio-Perez C. Tamborero D. Schroeder M. P. Antolín A. A. Deu-Pons J. Perez-Llamas C.. (2015). In silico prescription of anticancer drugs to cohorts of 28 tumor types reveals targeting opportunities. Cancer Cell 27, 382–396. 10.1016/j.ccell.2015.02.007 25759023

[B44] Sanz-Pamplona R. Lopez-Doriga A. Paré-Brunet L. Lázaro K. Bellido F. Alonso M. H.. (2015). Exome sequencing reveals amer1 as a frequently mutated gene in colorectal cancer. Clin. Cancer Res. 21, 4709–4718. 10.1158/1078-0432.CCR-15-0159 26071483 PMC4609254

[B45] Tamborero D. Gonzalez-Perez A. Lopez-Bigas N. (2013). Oncodriveclust: exploiting the positional clustering of somatic mutations to identify cancer genes. Bioinformatics 29, 2238–2244. 10.1093/bioinformatics/btt395 23884480

[B46] Tokheim C. J. Papadopoulos N. Kinzler K. W. Vogelstein B. Karchin R. (2016). Evaluating the evaluation of cancer driver genes. Proc. Natl. Acad. Sci. U.S.A. 113, 14330–14335. 10.1073/pnas.1616440113 27911828 PMC5167163

[B47] Uusitalo E. Kallionpää R. A. Kurki S. Rantanen M. Pitkäniemi J. Kronqvist P.. (2017). Breast cancer in neurofibromatosis type 1: overrepresentation of unfavourable prognostic factors. Br. J. Cancer 116:211. 10.1038/bjc.2016.403 27931045 PMC5243991

[B48] Velmurugan K. Varghese R. Fonville N. Garner H. (2017). High-depth, high-accuracy microsatellite genotyping enables precision lung cancer risk classification. Oncogene 36:6383. 10.1038/onc.2017.256 28759038 PMC5701090

[B49] Vogelstein B. Papadopoulos N. Velculescu V. E. Zhou S. Diaz L. A. Kinzler K. W. (2013). Cancer genome landscapes. Science 339:1546–1558. 10.1126/science.1235122 23539594 PMC3749880

[B50] Wang H. Shen Q. Zhang X. Yang C. Cui S. Sun Y.. (2017). The long non-coding rna xist controls non-small cell lung cancer proliferation and invasion by modulating mir-186-5p. Cell. Physiol. Biochem. 41, 2221–2229. 10.1159/000475637 28448993

[B51] Wang X.-W. Zhang Y.-J. (2014). Targeting mtor network in colorectal cancer therapy. World J. Gastroenterol. 20:4178. 10.3748/wjg.v20.i15.4178 24764656 PMC3989954

[B52] Wong W. C. Kim D. Carter H. Diekhans M. Ryan M. C. Karchin R. (2011). Chasm and snvbox: toolkit for detecting biologically important single nucleotide mutations in cancer. Bioinformatics 27, 2147–2148. 10.1093/bioinformatics/btr357 21685053 PMC3137226

[B53] Yates B. Braschi B. Gray K. A. Seal R. L. Tweedie S. Bruford E. A. (2016). Genenames. org: the hgnc and vgnc resources in 2017. Nucleic Acids Res. 45, D619–D625. 10.1093/nar/gkw1033 27799471 PMC5210531

[B54] Yu J. Wu W. Liang Q. Zhang N. He J. Li X.. (2016). Disruption of ncoa2 by recurrent fusion with lactb2 in colorectal cancer. Oncogene 35:187. 10.1038/onc.2015.72 25823027 PMC4717154

[B55] Yu Y. Liu D. Liu Z. Li S. Ge Y. Sun W.. (2018). The inhibitory effects of col1a2 on colorectal cancer cell proliferation, migration, and invasion. J. Cancer 9:2953. 10.7150/jca.25542 30123364 PMC6096367

[B56] Zheng X. Zhou C. Cheng H. Hu T. Liu H. Liu X. (2017). ELMO1 promotes metastasis in colorectal cancer cells via activation of MAPK/ERK signaling pathway. Cancer Res. 77(13 Suppl.):4849 10.1158/1538-7445.AM2017-4849

